# Development of a multiplex reverse transcription-quantitative PCR (qPCR) method for detecting common causative agents of swine viral diarrhea in China

**DOI:** 10.1186/s40813-024-00364-y

**Published:** 2024-03-05

**Authors:** Wenbo Song, Yixue Feng, Jiali Zhang, Danni Kong, Jie Fan, Mengfei Zhao, Lin Hua, Jinmei Xiang, Xibiao Tang, Shaobo Xiao, Zhong Peng, Bin Wu

**Affiliations:** 1 National Key Laboratory of Agricultural Microbiology, College of Veterinary Medicine, Huazhong Agricultural University, 430070 Wuhan, China; 2 Hubei Hongshan Laboratory, 430070 Wuhan, China; 3 Frontiers Science Center for Animal Breeding and Sustainable Production, The Cooperative Innovation Center for Sustainable Pig Production, 430070 Wuhan, China; 4 Animal Disease Diagnosis Center of Wuhan Keqian Biology Co., Ltd, 430070 Wuhan, China; 5 Hubei Vocational College of Bio-Technology, 430070 Wuhan, China

**Keywords:** TaqMan multiplex qPCR method, PEDV, TGEV, PDCoV, RVA, Swine viral diarrhea, Detection

## Abstract

**Background:**

Diarrheal diseases caused by viral agents have led to a great morbidity, mortality, and economic loss in global pig industry. Porcine epidemic diarrhea virus (PEDV), transmissible gastroenteritis virus (TGEV), porcine deltacoronavirus (PDCoV), and group A porcine rotavirus (RVA) are main causative agents of swine viral diarrhea with similar clinical signs on Chinese farms and their co-infection is also common. However, it is still lack of a convenient method to detect these four agents.

**Methods:**

A TaqMan multiplex qPCR method was developed to detect PEDV, TGEV, PDCoV, and RVA, simultaneously. This method was then applied to investigate 7,342 swine fecal samples or rectal swabs, as well as 1,246 swine intestinal samples collected from 2075 farms in China in 2022.

**Results:**

Minimum detection limits of this method were 3 copies/µL for PEDV, 4 copies/µL for TGEV, 8 copies/µL for RVA, and 8 copies/µL for PDCoV, suggesting a good sensitivity. No signals were observed by using this method detecting other viral agents commonly prevalent in pigs, which is suggestive of a good specificity. Application of this method on investigating clinical samples demonstrated a relatively high positive rate for PEDV (22.21%, 1907/8588) and RVA (44.00%, 3779/8588). In addition, co-infection between PEDV and RVA was observed on 360 investigated farms, accounting for 17.35% (360/2075) of the farms where co-infection events were screened.

**Conclusions:**

A TaqMan multiplex qPCR method targeting PEDV, TGEV, PDCoV, and RVA was developed in this study. This method demonstrated a good specificity and sensitivity on investigating these four common viruses responsible for viral diarrhea on Chinese pig farms, which represents a convenient method for the monitoring and differential diagnosis of swine viral diarrhea.

**Supplementary Information:**

The online version contains supplementary material available at 10.1186/s40813-024-00364-y.

## Background

Porcine epidemic diarrhea (PED) is an acute diarrheal disease caused by porcine epidemic diarrhea virus (PEDV) and has caused big economic losses to global pig industry [1]. In China, a new variant of PEDV with higher mortalities emerged in 2010, which makes the disease prevention and control more complex, and PED remains one of the threats to the pig industry in this largest pig rearing country in the world [2]. Transmissible gastroenteritis virus (TGEV) is also a porcine enteropathogenic coronavirus that can cause severe diarrhea and death in piglets [3]. In general, piglets under two weeks of age are most susceptible to TGEV, but this virus can still cause diarrhea and loss of appetite in old pigs [4]. TGEV can be also detected in pigs that have recovered from the infection [5].

In addition to PEDV and TGEV, porcine deltacoronavirus (PDCoV) is a newly emerged pig enteropathogenic coronavirus that can replicate in small intestinal cells and cause vomiting and watery diarrhea in piglets [6]. Pathological changes similar to those observed after PEDV and TGEV infection can also be seen in the intestines of pigs infected with PDCoV [7]. Porcine rotavirus (RV) can cause acute gastroenteritis in suckling and weaned piglets and suppress the immune system, leading to growth retardation and increased mortality in piglets [8]. Rotaviruses are classified into 10 groups or species (RVA-RVJ), based on the amino acid sequence of the structural protein, VP6. RVA is currently the most common pathogen causing clinical diarrhea in piglets.

It should be noted that clinical symptoms caused by above-mentioned four viruses are similar and those four agents are frequently associated with mixed infections on pig farms. Therefore, rapid diagnosis plays a crucial role in controlling porcine viral diarrhea. Currently, molecular diagnostic techniques such as Reverse Transcription-Polymerase Chain Reaction (RT-PCR), Reverse Transcription-Quantitative Polymerase Chain Reaction (qPCR), and Reverse Transcription Loop-Mediated Isothermal (RT-LAMP), are commonly used for pathogen detection [9–11]. However, it is still lack of a convenient method to detect the above-mentioned four agents. In this study, a multiplex TaqMan RT-qPCR detection method was developed to simultaneously identify and diagnose PEDV, TGEV, PDCoV, and RVA. This method meets the need for rapid diagnosis of porcine viral diarrhea in farms and laboratories and has been applied to the detection of clinical samples.

## Methods

### Primers, probes, and plasmids

Primers and probes used in this study are listed in Table 1. Standard plasmids were prepared as follows: plasmids containing the full-length of M gene from PEDV-AJ1102 (GenBank accession no. JX188454.1; 215.995 ng/µl), the full-length of M gene from TGEV-TH-98 (GenBank accession no. KU729220; 165.405 ng/µl), the full-length of NSP5 gene from RVA-HB-7 (GenBank accession no. MZ165432; 202.076 ng/µl), and the full-length of N gene from PDCoV-TS12019 (GenBank accession no. MT663769 233.108 ng/µl) were synthesized. PCR amplification was performed using pUC57 vector universal sequencing primers (M13R: 5’-CAGGAAACAGCTATGACC-3’; M13F: 5’-TGTAAAACGACGGCCAGT-3’) to verify the correctness of the synthesized sequence. Plasmid concentration was measured using Microvolume UV-Vis Spectrophotometers (Nano Drop One, Thermo Scientific).

**Table 1:** The primer used in this study

Virus	Primer	Sequence	Position	Product Size	Target gene
PEDV	PEDV-F	5’-GGTTGCTACTGGCGTACAGGTA-3’	26,148–26,169	105 bp	M gene
	PEDV-R	5’-GAAGCATTGACTGAACGACCAACA-3’	26,229–26,252		
	PEDV-P	5’-FAM-TCGTCACAGTCGCCAAGGCCACTACAACA-BHQ1-3’	26,186–26,213		
TGEV	TGEV-F	5’-GCGTTAGTGCATTAGGAAGAAGCTA-3’	26,562–26,586	91 bp	M gene
	TGEV-R	5’-GCGTACAAATTCCCTGAAAGCAAAG-3’	26,628–26,652		
	TGEV-P	5’-HEX-CCTCTCGAAGGTGTGCCAACTGGTGTCACTCT-BHQ1-3’	26,594–26,625		
RVA	RVA-F	5’-TGAATCGTCTTCTACAACGTCAAC-3’	81–104	147 bp	NSP5
	RVA-R	5’-TCGTTTGAAGCAGAATCAGATGG-3’	205–227		
	RVA-P	5’-ROX-CTCTGGAGACTTCGACAACAT-MGB-3’	175–195		
PDCoV	PDCoV-F	5’-GGTCGTTAACCAGACCTATGAG-3’	24,847–24,868	177 bp	N gene
	PDCoV-R	5’-GCTGCTGATTCCTGCTTTATCTC-3’	25,001–25,023		
	PDCoV-P	5’-CY5-CCAACTAAGGACAAGAAGCCTGACA-BHQ3-3’	24,881–24,905		
PEDV	PEDV-F1	5’-GGCGGTTCTTTTCAAAATTTAAT-3’	20,731–20,753	1981 bp	Partial S1 gene
	PEDV-R1	5’-GCACCACTAGTGACATTCTTAAA-3’	22,689–22,711		
RVA	RVA-F1	5’-GGCTTTAAAAGAGAGAATTTC-3’	1–21	1060 bp	VP7 gene
	RVA-R1	5’-GGTCACATCATACAATTC-3’	1043–1060		

### Optimization of reaction system

Optimization of the reaction system was conducted using the chessboard titration method, as described previously [12]. A 20.0 µL reaction system (2 × One Step RT-PCR buffer, 10.0 µL; enzyme premix, 1.0 µL; standard plasmid, 1.0 µL; gradient increase of primer and probe amount from 0.15 µM to 0.3 µM with increments of 0.025 µM, and DEPC water) was prepared and the reaction was performed in a Bio-Rad CFX96 Real-Time PCR System (Bio-Rad, Hercules, CA). The reaction conditions were: reverse transcription at 55 °C for 30 min; pre-denaturation at 95 °C for 30 s; denaturation at 95 °C for 5 s, annealing at 60 °C for 30 s, for 40 cycles, with fluorescence signal collection at 60 °C.

### Establishment of standard curve

Standard plasmids were serially diluted (10-folds, from 10^8^ to 10^0^ copies/µL), and amplification was performed using the optimized reaction system for standard samples of different dilutions. DNA copies were calculated using the following formula: copies/µL = [plasmid concentration (ng/µL) × 10^− 9^ × 6.02 × 10^23^ (copies/mol)]/(DNA length × 660) [13]. Standard curves were drawn based on the results.

### Specificity test

Nucleic acids from porcine reproductive and respiratory syndrome virus (PRRSV), classical swine fever virus (CSFV), porcine circovirus type 2 (PCV2), swine acute diarrhea syndrome coronavirus (SADS-CoV), Bocavirus, *Escherichia coli* (*E. coli*), *Lawsonia intracellularis* (LI), and/or *Clostridium perfringens* were extracted and used for testing the specificity of the detection method with the optimized reaction scheme.

### Sensitivity test

The standard plasmids with concentrations ranging from 10^8^ to 10^0^ copies/µL were amplified using the optimal reaction scheme, and the lowest detection limit copy number of the detection method was determined.

### Comparison with commercial kit

To verify the accuracy and reproductivity, the detection efficacy of the method developed in this study was compared with a commercial PEDV/TGEV/PRVA/PDCoV nucleic acid-detection kit (Vipotion Biotechnology, Guangzhou, China). Samples used for the evaluation included known positive samples which were diluted ten times and/or randomly-selected clinical samples (*n* = 80). Different samples were detected using the method developed in this study and the purchased commercial kit respectively.

### Sample detection using the multiplex qRT-PCR method

From January 2022 to December 2022, a total of 7,342 swine fecal samples or rectal swabs, 1,246 intestinal tissues were collected from various pig farms in China. Samples were treated as follows: (1) fecal samples were mixed thoroughly with an equal volume of physiological saline, followed by centrifugation at 8,000 rpm for 2 min to collect the supernatants; (2) swabs were maintained in tubes containing 500 µL of physiological saline and shaken vigorously, followed by centrifugation at 8,000 rpm for 2 min to collect the supernatants; (3) intestinal samples (50 $\sim$ 100 mg) were homogenized in 1 mL of physiological saline, followed by centrifugation at 8,000 rpm for 2 min to collect the supernatants. Afterwards, viral RNAs were extracted using a commercial nucleic acid extraction kit (Zhongkebio, Nanjing, China) according to the manufacturer’s instructions, which were then reverse to cDNAs and used as the templates for investigating the above-mentioned for viral agents by the method developed in this study.

### Evolutionary analysis

We selected PEDV and RVA positive samples and amplified the partial S1 gene of PEDV as well as the VP7 gene of RVA using primers PEDV-F1/PEDV-R1, RVA-F1/RVA-R1 by PCR, respectively. Reactions were performed in a 25 µL mixture containing 12.5 µL of 2×One Step Buffer, 1 µL of Primer Script One Step Enzyme Mix, 1 µL of each forward and reverse primers (10 µM), and 7.5 µL of nucleic acid-free H_2_O, and 2 µL of RNA template. Reaction conditions were as follows: 42 °C for 30 min; 94 °C for 5 min; followed by 30 cycles of 94 °C for 30 s, 53 °C for 30 s, 72 °C for 2 min; and extension at 72 °C for 7 min. PCR products were sent to Shanghai Bioengineering Company for Sanger sequencing, and the sequencing results were subjected to genetic evolution analysis using MEGA6 software [14].

## Results

### Optimization of multiplex qPCR reaction system and establishment of standard curves

**Fig. 1:**
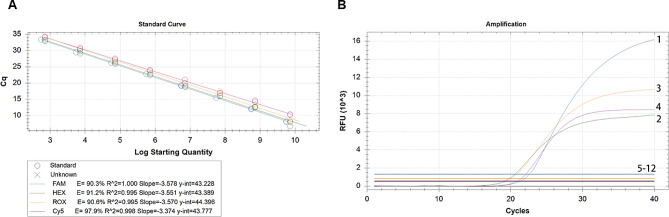
**Assessment of linear relationship and specificity of the TaqMan multiplex qPCR method developed in this study. (A)** Linear relationship between the *Ct* values and the copy numbers of standard plasmids; **(B)** Amplification curves given by the multiplex method on detecting different agents; 1–4: Nucleic acids of PEDV, TGEV, RVA, PDCoV; 5–12: Nucleic acids of porcine reproductive and respiratory syndrome virus (PRRSV), classical swine fever virus (CSFV), porcine circovirus type 2 (PCV2), swine acute diarrhea syndrome coronavirus (SADS-CoV), bocavirus, *Escherichia coli*, *Lawsonia intracellularis*, and/or *Clostridium perfringens*

The optimal primer and probe concentrations for qRT-PCR reactions were determined using 10^6^ copies/µL of standard plasmid as a template, along with the comprehensive consideration of the amplification C*t* value, fluorescence intensity, and amplification curve (Tables S1–S4 and Fig. S1 in supplementary information). The optimal final concentrations of primers and probes were 0.225 µM (primers) and 0.15 µM (probe) for detecting PEDV, 0.225 µM (primers) and 0.2 µM (probe) for detecting TGEV, 0.25 µM (primers) and 0.25 µM (probe) for detecting RVA, and 0.2 µM (primers) and 0.25 µM (probe) for detecting PDCoV, respectively. The correlation coefficients (*R*^2^) of PEDV (FAM), TGEV (HEX), RVA (ROX), and PDCoV (Cy5) were 1.000, 0.995, 0.995, and 0.998, respectively (Fig. 1A). This indicates that the detection method established in this study has a good linear relationship, and the amplification efficiency is between 90.3% and 97.9%.

### Assessment of specificity and sensitivity

Specificity was assessed by applying the established multiplex qRT-PCR method to detect the nucleic acids of PRRSV, CSFV, PCV2, SADS-CoV, *E. coli*, LI, *C. perfringens*, PEDV, TGEV, RVA, or PDCoV. The results showed that the method only produced typical amplification curves in PEDV, TGEV, RVA, and PDCoV, and there was no cross-reaction between the four pathogens (with duplicate wells set up) (Fig. 1B). The lowest detectable copy numbers of the method developed in this study were 3 copies/µL for PEDV, 4 copies/µL for TGEV, 8 copies/µL for RVA, and 8 copies/µL for PDCoV, respectively. Evaluation using standard plasmids indicated a good repeatability for the method developed in this study, with coefficients of variation between 0.20 $\sim$ 1.09 (Table 2). The agreement rates between the method developed in this study and the commercial kit on detecting clinical samples (*n* = 80) were 97.5% (PEDV), 95.0% (TGEV), 86.3% (RVA), and 98.8% (PDCoV), respectively (Table 3).

**Table 2:** Sensitivity and repeatability of multiplex qRT-PCR

Plasmid	Copy number	Ct(mean ± S.D.)	CV%	Plasmid	Copy number	Ct(mean ± S.D.)	CV%	Plasmid	Copy number	Ct(mean ± S.D.)	CV%	Plasmid	Copy number	Ct(mean ± S.D.)	CV%
PEDV	5.0 × 10^9^	8.09 ± 0.02	0.23%	TGEV	5.0 × 10^9^	8.16 ± 0.03	0.42%	RV	5.0 × 10^9^	8.30 ± 0.06	0.74%	PDCoV	5.0 × 10^9^	8.03 ± 0.09	1.09%
	5.0 × 10^8^	11.85 ± 0.05	0.45%		5.0 × 10^8^	11.79 ± 0.03	0.28%		5.0 × 10^8^	11.85 ± 0.05	0.45%		5.0 × 10^8^	11.52 ± 0.07	0.60%
	5.0 × 10^7^	15.38 ± 0.04	0.27%		5.0 × 10^7^	15.36 ± 0.05	0.32%		5.0 × 10^7^	15.42 ± 0.03	0.22%		5.0 × 10^7^	14.87 ± 0.06	0.43%
	5.0 × 10^6^	19.19 ± 0.04	0.22%		5.0 × 10^6^	18.92 ± 0.02	0.13%		5.0 × 10^6^	19.11 ± 0.05	0.26%		5.0 × 10^6^	18.35 ± 0.07	0.38%
	5.0 × 10^5^	22.74 ± 0.05	0.22%		5.0 × 10^5^	22.46 ± 0.05	0.24%		5.0 × 10^5^	22.61 ± 0.06	0.27%		5.0 × 10^5^	21.83 ± 0.10	0.34%
	5.0 × 10^4^	26.36 ± 0.06	0.22%		5.0 × 10^4^	26.1 ± 0.04	0.17%		5.0 × 10^4^	26.21 ± 0.07	0.29%		5.0 × 10^4^	25.31 ± 0.06	0.24%
	5.0 × 10^3^	29.85 ± 0.07	0.23%		5.0 × 10^3^	29.57 ± 0.06	0.19%		5.0 × 10^3^	29.85 ± 0.08	0.26%		5.0 × 10^3^	28.72 ± 0.08	0.25%
	5.0 × 10^2^	33.51 ± 0.08	0.23%		5.0 × 10^2^	33.25 ± 0.07	0.22%		5.0 × 10^2^	33.34 ± 0.07	0.22%		5.0 × 10^2^	32.25 ± 0.08	0.24%
	5.0 × 10^1^	36.03 ± 0.07	0.20%		5.0 × 10^1^	36.64 ± 0.10	0.26%		5.0 × 10^1^	36.77 ± 0.09	0.24%		5.0 × 10^1^	35.64 ± 0.12	0.20%
	10	36.70 ± 0.13	0.35%		10	36.78 ± 0.14	0.39%		10	37.10 ± 0.12	0.32%		10	36.42 ± 0.17	0.46%
	5	37.36 ± 0.19	0.50%		5	37.41 ± 0.10	0.26%		9	37.70 ± 0.15	0.39%		9	37.08 ± 0.17	0.47%
	4	37.30 ± 0.23	0.62%		4	37.72 ± 0.12	0.32%		8	37.70 ± 0.14	0.36%		8	37.61 ± 0.13	0.35%
	3	37.52 ± 0.26	0.69%		3	None	None		5	None	None		5	None	None
	1	None	None		1	None	None		1	None	None		1	None	None

**Table 3:** Agreement rates between detecting clinical samples using the commercial kit and the method developed in this study

		Commercial kit			Agreement rate
		Positive	Negative	Total	
**PEDV**					
The method developed in this study	Positive	30	1	31	97.5%
	Negative	1	48	49	
	Total	31	49	80	
**TGEV**					
The method developed in this study	Positive	17	3	20	95.0%
	Negative	1	59	60	
	Total	18	62	80	
**RVA**					
The method developed in this study	Positive	27	10	37	86.3%
	Negative	1	42	43	
	Total	29	51	80	
**PDCoV**					
The method developed in this study	Positive	32	1	33	98.8%
	Negative	0	47	47	
	Total	32	48	80	

### Clinical sample investigation

**Fig. 2:**
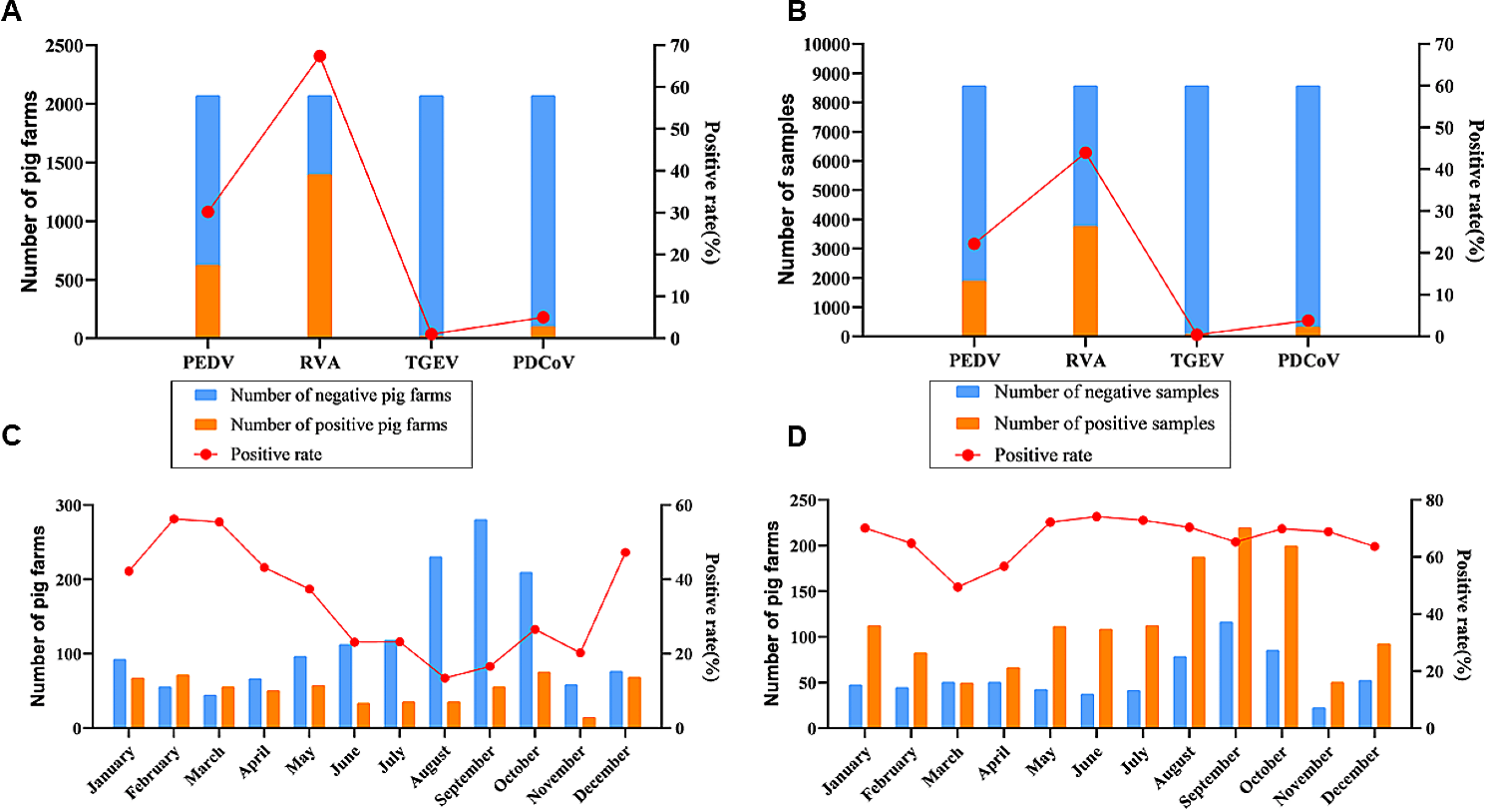
**Investigation of clinical samples from pig farms in China using the TaqMan multiplex qPCR method. (A)** Farm positivity of PEDV, TGEV, RVA, and PDCoV investigated using the TaqMan multiplex qPCR method; **(B)** Sample positivity of PEDV, TGEV, RVA, and PDCoV investigated using the TaqMan multiplex qPCR method; **(C)** Positive detection rate of PEDV in different months on different pig farms; **(D)** Positive detection rate of RVA in different months on different pig farms; PEDV: porcine epidemic diarrhea virus, TGEV: transmissible gastroenteritis virus, PDCoV: porcine deltacoronavirus, RVA: group A porcine rotavirus

From January to December 2022, a total of 8588 samples of intestinal contents and feces from dead pigs and rectal swabs from diarrheal pigs from 29 provinces of China were collected for PEDV, RVA, PDCoV, and/or TGEV investigation by the multiplex qPCR method generated in this study. The results revealed that 30.22% (627/2075) of the detected farms were positive for PEDV, 67.42% (1399/2075) of the detected farms were positive for RVA, 5.01% (104/2075) of the detected farms were positive for PDCoV, and 1.01% (21/2075) of the detected farms were positive for TGEV, with an overall detection rate of 22.21% (1907/8588; PEDV), 44.00% (3957/8588; RVA), 3.85% (331/8588; PDCoV), and 0.41% (35/8588; TGEV) for the four agents, respectively (Fig. 2A and B). Monthly, the positive detection rate of PEDV on farm-level was relatively low in June, July, and August, indicating that summer is the trough period for PEDV prevalence in China (Fig. 2C). However, the detection of RVA demonstrated low-level of season-preference in our investigation (Fig. 2D). The farm-level positive detection rate of RVA was higher than 49% in every month. The lowest rate is 49.50% (50/101) in March, while the highest rate reaches 74.15% (109/147) in June. This epidemic trend shows no seasonality, indicating that RVA has a high prevalence rate throughout the year and should draw the attention of pig farm breeders.

**Fig. 3:**
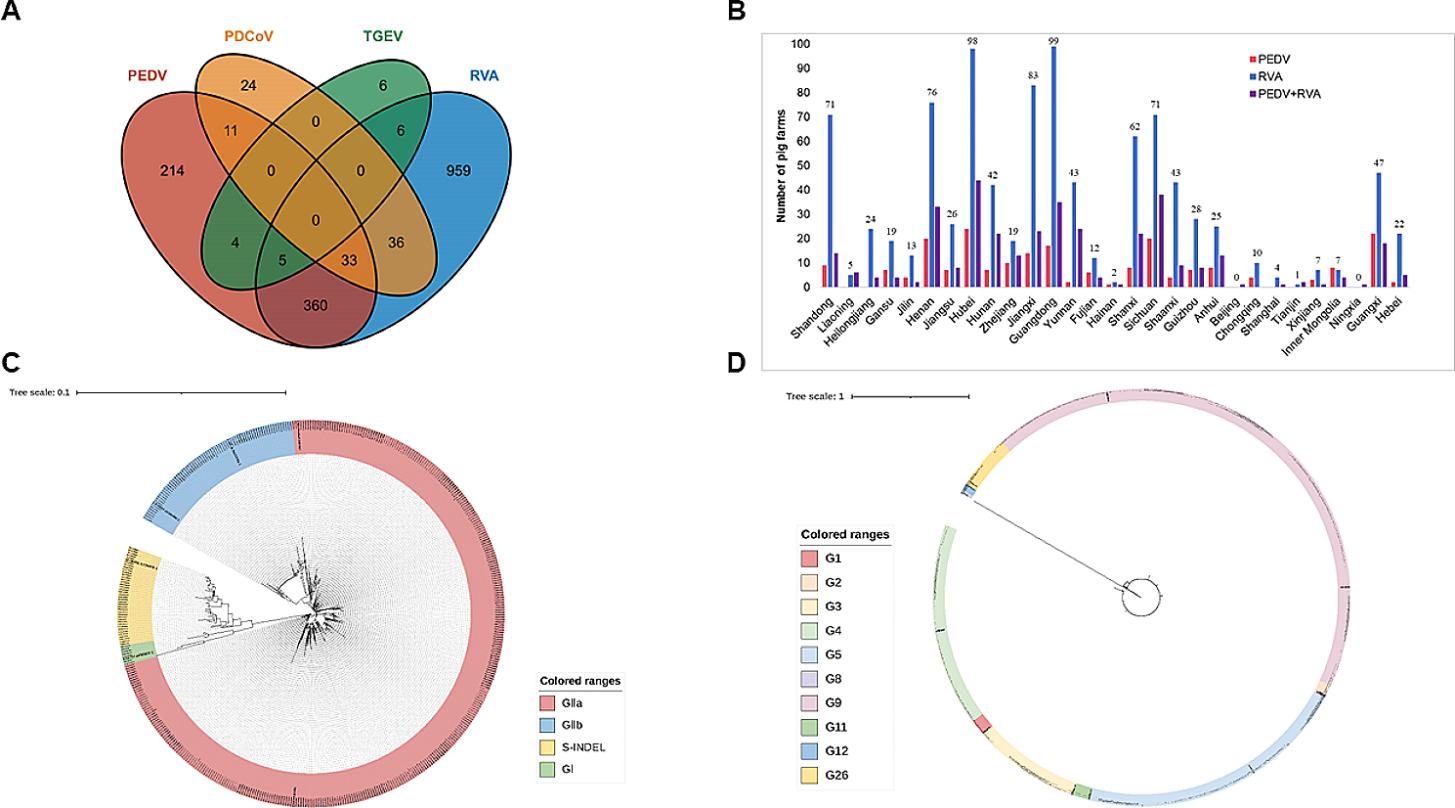
**Prevalent profile of PEDV, TGEV, RVA, and PDCoV on pig farms in China. (A)** A Venn diagram showing the prevalence of the four swine diarrhea viruses on farms in China; **(B)** A column chart displaying the provinces where pig farms with different profile of the four agents located; **(C)** Phylogenetic analysis of 460 PEDV characterized in this study based on the S1 gene; **(D)** Phylogenetic analysis of 861 RVA characterized in this study based on the VP7 gene; PEDV: porcine epidemic diarrhea virus, TGEV: transmissible gastroenteritis virus, PDCoV: porcine deltacoronavirus, RVA: group A porcine rotavirus

Next, we applied the developed multiplex qPCR method to investigate the profile of PEDV, RVA, TGEV, and PDCoV in samples from 2,075 Chinese pig farms. The results demonstrated that samples from 959, 214, 24, and 6 farms were only positive for RVA, PEDV, PDCoV, and TGEV, respectively (Fig. 3A). In addition, samples from 360 farms were positive for RVA and PEDV simultaneously, accounting for 17.35% (360/2075) of the total farms (Fig. 3A). These 360 farms were distributed in 28 Chinese provinces (Fig. 3B). Samples from 36, 11, 6, and 4 farms were simultaneously positive for RVA plus PDCoV, PEDV plus PDCoV, RVA plus TGEV, and PEDV plus TGEV, respectively (Fig. 3A). Notably, samples from 33 to 5 farms were simultaneously positive for PEDV plus RVA plus PDCoV, and PEDV plus RVA plus TGEV, respectively (Fig. 3A).

### Genotypes of diarrheal viruses on Chinese pig farms

To understand the genotypes of PEDV and RVA prevalent in China, Sanger sequencing was performed on PEDV-S1 genes from 1907 positive samples and RVA-VP7 genes from 3779 positive samples. This approach led to the collection of 460 PEDV-S1 sequences and 861 RVA-VP7 sequences. Phylogenetic analysis showed that there were four (sub-)genotypes characterized for PEDVs, including GI, GIIa, GIIb, and S-Indel (Fig. 3C; Table 4). The predominant PEDV (sub-)genotype was GIIa (74.78%, 344/460), followed by GIIb (15.22%, 70/460), S-Indel (8.48%, 39/460), and GI (1.52%, 7/460) (Fig. 3C; Table 3). There were ten (sub-)genotypes determined for RVAs, and G9 (45.18%, 389/861), G5 (21.84%, 188/861), and G4 (16.26%, 140/861) were the predominate types (Fig. 3D; Table 4). Additionally, G3 (8.59%, 74/861), G26 (4.30%, 37/861), G11 (1.28%, 11/861), and other types were also detected (Fig. 3D; Table 4).

**Table 4:** Analysis of sequencing results of PEDV and RVA

	PEDV				RVA									
Genotypes	GIIa	GIIb	S-Indel	GI	G9	G5	G4	G3	G26	G11	G1	G2	G8	G12
Number	344	70	39	7	389	188	140	74	37	11	10	7	1	4
Percentage	74.78%	15.22%	8.48%	1.52%	45.18%	21.84%	16.26%	8.59%	4.30%	1.28%	1.16%	0.81%	0.12%	0.46%

## Discussion

As leading causes of swine diarrhea, PEDV, RVA, PDCoV and/or TGEV are frequently characterized on worldwide pig farms [15, 16]. It is of clinical significance to develop rapid and accurate multiplex methods to differentiate these four agents from diarrheal causes as the symptoms induced by them are similar [11]. However, documented methods for investigation of these four agents simultaneously are still rare, and we therefore developed a multiplex qPCR method to investigate PEDV, TGEV, RVA, and PDCoV in this study. The envelope protein (M protein) and the nucleocapsid protein (N protein) are conserved proteins encoded by coronaviruses and both of them are widely used for the diagnosis of coronaviruses, including PEDV, TGEV, and PDCoV [17–19]. The nonstructural protein NSP5 is required for viroplasm formation and virus replication of Rotaviruses (RVs) [20, 21]. This protein is also a conserved RV protein [22, 23], and has been recognized a promising target for RV diagnosis in several studies [24, 25]. In agreement with these studies, we selected the M gene as the target gene used for detecting PEDV and TGEV, the N gene as the target for detecting PDCoV, and NSP5 as the target for detecting RVA in this study. The lowest detectable copy numbers of the method assessed using synthesized plasmids were lower than 10 copies/µL (3 copies/µL for PEDV, 4 copies/µL for TGEV, 8 copies/µL for RVA, and 8 copies/µL for PDCoV). These detection limits were similar to those (2.95 × 10^0^ copies/µL) in a documented multiplex qPCR which was developed to detect PEDV, TGEV, and PDCoV simultaneously [26]. These detection limits were much lower than those reported in another multiplex qPCR which was developed for detecting different types of PEDV (20 copies/µL for GI and 100 copies/µL for GII, 50 copies/µL for both RVA and RVC) [27]. These findings suggest the multiplex developed in this study possess a good sensitivity. In addition, our developed multiplex method did not provide any amplification curves during detecting other swine or swine diarrhea associated pathogens including PRRSV, CSFV, PCV2, SADS-CoV, *E. coli*, LI, and *C. perfringens*, suggesting a good specificity.

Application of the developed multiplex qPCR method on investigating clinical samples indicated a common condition of mixed infection associated with swine diarrhea on farms, which agrees well with the other studies [19, 28]. This common phenomenon highlights the clinical necessary and significance of developing a multiplex qPCR for the rapid diagnosis. The results of this study demonstrated that RVA, followed by PEDV, were the predominant agents responsible for swine diarrhea on Chinese pig farms. These findings agree well with those from the other published articles [8, 29]. Of particularly note is RVA, which displays an increasing trend of detection in pigs in China in recent years [8, 15]. While rotaviruses could be divided into ten different serogroups (A $\sim$ J), RVA has been recognized as the most important rotavirus in swine enteric diseases, with a significant economic impact on pig production [30]. This is also why the current study selected this agent as a target for the method development. However, a high detection rate of RVA presented in this study and the other studies highlights the important role of this agent in the occurrence of swine diarrhea on pig farms and its management and control should receive more attention. Our investigation revealed that the detection of TGEV was relatively low on Chinese pig farms, which agrees well with those from the other studies [15, 31]. It is notable that the detection rate of TGEV on pig farms in the US displayed a continuous decrease trend from 2008 to 2016 (as low as 0.1%) [32], and it also has a low detection rate on pig farms in the other countries [33]. These findings suggest that the prevalence of TGEV on worldwide pig farms is low, and this might be because the rapid spread of porcine respiratory coronavirus which is closely related to TGEV in the 1980s provides immunological cross-protection [34]. The results of this study indicated a role of PDCoV in the occurrence of swine diarrhea on farms, even though the detection rate of this agent is relatively low. As a newly emerged swine diarrhea associated coronavirus, PDCoV generally do not possess a detection rate as high as the other main diarrhea-causing viral agents on pig farms in many studies [28, 31, 35]. However, the impact of PDCoV should not be ignored due to the potential zoonosis of the virus [36].

Like the findings from the other reports [15, 37], our results presented in this study showed that PEDV type GII particularly GIIa was still the predominant genotypes on pig farms in China. However, our phylogenetic analysis revealed that the GIIa branches clearly comprised of a heterogeneous of distinct clades, and these clades have been assigned as novel genotypes such as GIIc or GIId in several studies [38, 39]. It remains to be clarified whether those “novel genotypes” should be acknowledged as more solid evidence is still necessary. In addition to GII strains, our investigation showed that approximately 8.48% (39/460) of the strains were characterized as S-INDEL. This value is similar to that (9.7%) characterized in the North America, and the emergence of the S-INDEL strain is believed to have possibility to make the PEDV epidemic more complex [40]. Our results presented in this study demonstrated a complex condition on the prevalence of RVA on Chinese pig farms, as evidenced by the characterization of 10 RVA genotypes. Among these genotypes, G9, G5, and G4 were the predominantly characterized genotypes, which agrees well with the other reports from both China and other countries [41, 42], indicating an active role these genotypes in promoting swine diarrhea.

In conclusion, we developed a TaqMan multiplex qPCR method to detect PEDV, TGEV, PDCoV, and RVA in this study. This method demonstrated a good specificity and sensitivity, and could be used as a convenient method for the monitoring and differential diagnosis of swine viral diarrhea. Applying this method, we investigated the profile of these four diarrhea-associated viruses on Chinese pig farms, and our results indicate a complex condition on the prevalence of swine diarrheal viruses.

### Electronic supplementary material

Below is the link to the electronic supplementary material.

Supplementary Material 1

Supplementary Material 2

Supplementary Material 3

Supplementary Material 4

Supplementary Material 5

## Data Availability

No datasets were generated or analysed during the current study.

## Abbreviations

CSFV: classical swine fever virus
LI: *Lawsonia intracellularis*
PCV2: porcine circovirus type 2
PDCoV: porcine deltacoronavirus
PEDV: porcine epidemic diarrhea virus
PRRSV: porcine reproductive and respiratory syndrome virus
qPCR: reverse transcription-quantitative PCR
RVA: porcine rotavirus A
SADS-CoV: swine acute diarrhea syndrome coronavirus
TGEV: transmissible gastroenteritis virus
