# Supplementary material for: Development of a multiplex reverse transcription-quantitative PCR (qPCR) method for detecting common causative agents of swine viral diarrhea in China
Source: Porcine Health Manag. 2024 Mar 5;10:12. doi: 10.1186/s40813-024-00364-y (PMC10916220; doi:10.1186/s40813-024-00364-y)
Supplement: Supplementary file 1 — Supplementary Material 1 [file 40813_2024_364_MOESM1_ESM.doc]

Table S1. Primer and probe optimization of PEDV (FAM)

| Primer  Probe | 0.150 μM | 0.175 μM | 0.200 μM | 0.225 μM | 0.250 μM | 0.300 μM |
| --- | --- | --- | --- | --- | --- | --- |
| 0.100 μM | 17.36 | 16.87 | 16.64 | 16.99 | 17.27 | 16.35 |
| 0.150 μM | 17.08 | 16.78 | 16.45 | **16.61** | 16.90 | 16.26 |
| 0.200 μM | 16.95 | 16.56 | 16.12 | 16.42 | 16.79 | 16.26 |
| 0.250 μM | 16.85 | 16.53 | 16.27 | 16.28 | 16.64 | 16.29 |
