# Supplementary material for: Development of a multiplex reverse transcription-quantitative PCR (qPCR) method for detecting common causative agents of swine viral diarrhea in China
Source: Porcine Health Manag. 2024 Mar 5;10:12. doi: 10.1186/s40813-024-00364-y (PMC10916220; doi:10.1186/s40813-024-00364-y)
Supplement: Supplementary file 2 — Supplementary Material 2 [file 40813_2024_364_MOESM2_ESM.doc]

Table S2. Primer and probe optimization of TGEV (HEX)

| Primer  Probe | 0.150 μM | 0.175 μM | 0.200 μM | 0.225 μM | 0.250 μM | 0.300 μM |
| --- | --- | --- | --- | --- | --- | --- |
| 0.100 μM | 14.27 | 14.03 | 14.20 | 14.28 | 14.19 | 14.60 |
| 0.150 μM | 13.66 | 13.84 | 13.78 | 13.81 | 13.99 | 14.54 |
| 0.200 μM | 13.74 | 13.61 | 13.58 | **13.38** | 13.94 | 13.75 |
| 0.250 μM | 13.29 | 13.35 | 13.91 | 13.82 | 13.95 | 13.84 |
