# Supplementary material for: Development of a multiplex reverse transcription-quantitative PCR (qPCR) method for detecting common causative agents of swine viral diarrhea in China
Source: Porcine Health Manag. 2024 Mar 5;10:12. doi: 10.1186/s40813-024-00364-y (PMC10916220; doi:10.1186/s40813-024-00364-y)
Supplement: Supplementary file 3 — Supplementary Material 3 [file 40813_2024_364_MOESM3_ESM.doc]

Table S3. Primer and probe optimization of RVA (ROX)

| Primer  Probe | 0.150 μM | 0.175 μM | 0.200 μM | 0.225 μM | 0.250 μM | 0.300 μM |
| --- | --- | --- | --- | --- | --- | --- |
| 0.100 μM | 18.77 | 18.44 | 18.35 | 18.34 | 18.40 | 18.61 |
| 0.150 μM | 18.31 | 18.29 | 18.37 | 18.30 | 18.24 | 18.22 |
| 0.200 μM | 18.33 | 18.31 | 18.34 | 18.13 | 17.89 | 18.11 |
| 0.250 μM | 18.16 | 18.07 | 18.03 | 17.84 | **17.83** | 17.63 |
