# Supplementary material for: Development of a multiplex reverse transcription-quantitative PCR (qPCR) method for detecting common causative agents of swine viral diarrhea in China
Source: Porcine Health Manag. 2024 Mar 5;10:12. doi: 10.1186/s40813-024-00364-y (PMC10916220; doi:10.1186/s40813-024-00364-y)
Supplement: Supplementary file 4 — Supplementary Material 4 [file 40813_2024_364_MOESM4_ESM.doc]

Table S4. Primer and probe optimization of PDCoV (Cy5)

| Primer  Probe | 0.150 μM | 0.175 μM | 0.200 μM | 0.225 μM | 0.250 μM | 0.300 μM |
| --- | --- | --- | --- | --- | --- | --- |
| 0.100 μM | 14.65 | 14.27 | 14.62 | 14.38 | 14.04 | 14.07 |
| 0.150 μM | 14.38 | 14.04 | 14.21 | 14.46 | 13.93 | 13.62 |
| 0.200 μM | 14.41 | 14.03 | 13.58 | 13.40 | 14.01 | 14.15 |
| 0.250 μM | 13.95 | 14.73 | **13.06** | 13.34 | 13.41 | 13.48 |
