# Supplementary material for: Development of a multiplex reverse transcription-quantitative PCR (qPCR) method for detecting common causative agents of swine viral diarrhea in China
Source: Porcine Health Manag. 2024 Mar 5;10:12. doi: 10.1186/s40813-024-00364-y (PMC10916220; doi:10.1186/s40813-024-00364-y)
Supplement: Supplementary file 5 — Supplementary Material 5 [file 40813_2024_364_MOESM5_ESM.doc]

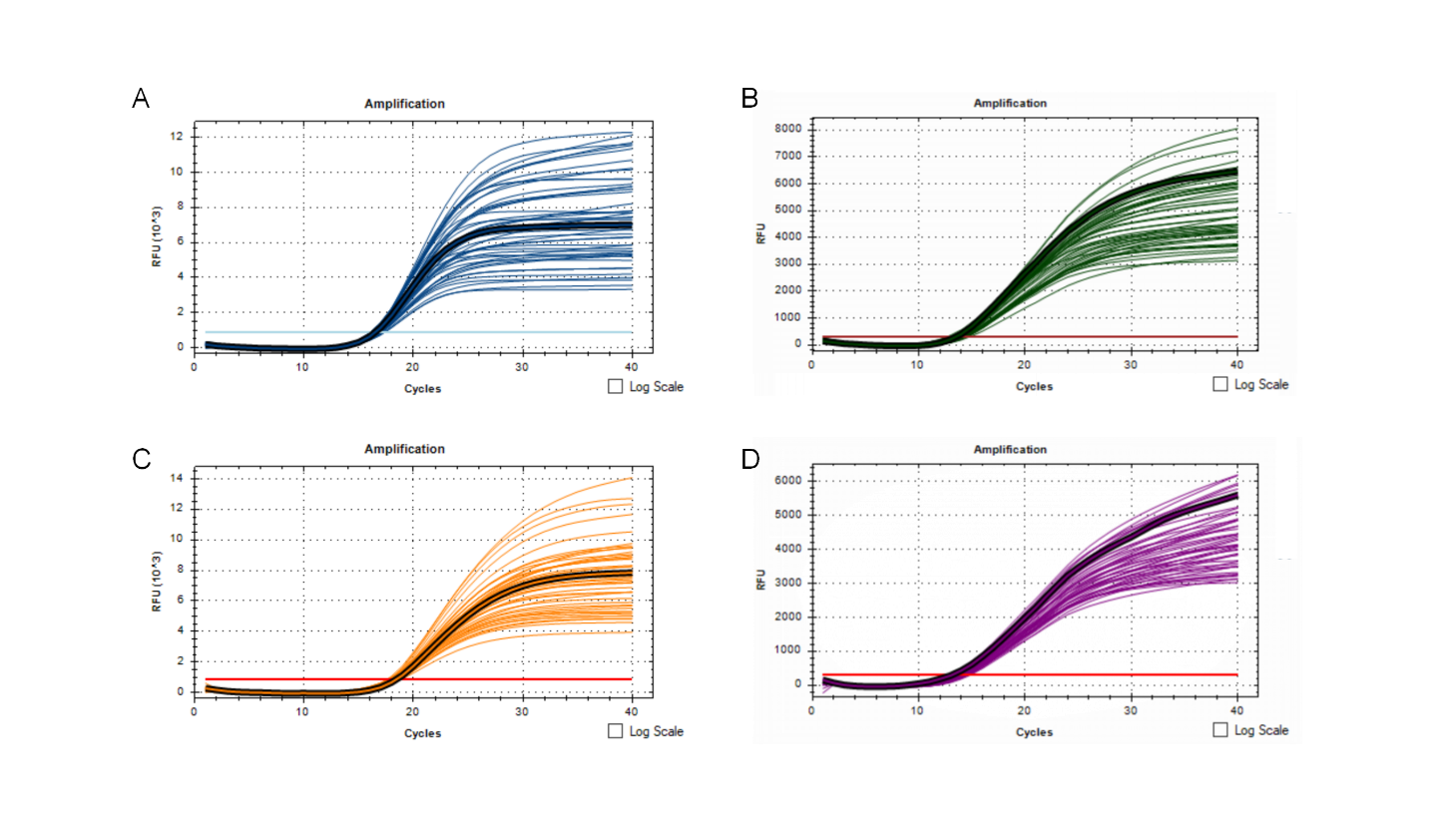


**Fig. S1.** Amplification curves generated under different concentrations of primers and probes on detecting PEDV (panel A), TGEV (panel B), RVA (panel C), and PDCoV (panel D).
